# Human and Animal Infections with *Mycobacterium microti,* Scotland

**DOI:** 10.3201/eid1312.061536

**Published:** 2007-12

**Authors:** Francis Xavier Emmanuel, Amie-Louise Seagar, Christine Doig, Alan Rayner, Pauline Claxton, Ian Laurenson

**Affiliations:** * Scottish Mycobacteria Reference Laboratory, Edinburgh, Scotland, United Kingdom

**Keywords:** Tuberculosis, zoonoses, domestic animals, wild animals, zoo, immunocompromised patients, molecular epidemiology, Scotland, dispatch

## Abstract

During 1994–2005, we isolated *Mycobacterium microti* from 5 animals and 4 humans. Only 1 person was immunocompromised. Spoligotyping showed 3 patterns: vole type, llama type, and a new variant llama type.

Naturally occurring mycobacteria that are part of the *Mycobacterium tuberculosis* complex include *M. tuberculosis, M. bovis, M. caprae, M. africanum, M. microti,* and *M. pinnipedii*. Although these species show remarkable genetic homology, there are notable phenotypic differences, particularly in their relative pathogenicity for different mammalian species.

Tuberculosis in wild rodents was first studied in 1937 as part of an investigation of cyclical changes in the population density of voles (*1*). Field voles, bank voles, wood mice, and shrews are particularly susceptible to infection with *M. microti* (*2*). However, other small mammals such as guinea pigs, rabbits, mice, and rats are resistant to *M.microti* infection, even at high doses of infection. More recently, sporadic cases have been described in larger mammals (*3*–*6*).

There have been only 6 published reports of human infections, comprising 13 patients in total (*7*–*11*). Salient information from these reports is summarized in Table 1.

**Table 1:** Summary of all reported cases of human infections with Mycobacterium microti*

Case-patient no.	Ref	Age, sex, country	Immune status	Infection site	Animal contact	Laboratory findings	Outcome
1	(*8*)	48 y, M, Germany	HIV positive	Lung	None	Llama type. Good growth in liquid medium. Poor on pyruvate. Drug susceptible. Curved bacilli.	Cured
2	(*7*,*10*)	39 y, M, the Netherlands	HIV positive	Lung; lymph nodes	House mice	Cultures negative; curved bacilli in sputum. Vole type on direct spoligotyping.	Cured after prolonged therapy†
3	(*7*)	12 y, M, the Netherlands	Renal transplant	Lung meninges	None	Vole type. Other details unavailable.	Cured
4	(*7*)	41 y, M, the Netherlands	Renal transplant	Peritoneal	Wild small rodents	Vole type. Other details unavailable.	Died despite therapy
5	(*7*)	34 y, M, the Netherlands	Normal	Lung	Lived in mobile home	Vole type. Other details unavailable.	Cured
6	(*9*)	53 y, M, Germany	Normal	Lung	None	Llama type. AFB film negative. Liquid culture better than pyruvate agar. No growth on normal egg media. Fully drug susceptible. Noncurved bacilli.	Cured
7	(*9*)	58 y, M, Germany	Diabetic	Lung	None	Vole type. Growth on liquid culture only (poor). Susceptibility not done. Noncurved bacilli.	Cured
8	(*5*)	Not known; England or Wales	Not known	Not known	Not known	Llama type.	Not known
9 to 12	(*5*)	Not known; England or Wales	Not known	Not known	Not known	Vole type.	Not known
13	(*11*)	69 y, sex not known, Germany	Normal	Abdominal/ miliary	Not known	Vole type. Primary culture in liquid. Subculture in solid agar.	Died, despite appropriate therapy

*Ref, reference; M, male; AFB, acid-fast bacilli. †Three household contacts were found to be tuberculin positive.

*M. microti* has been used in extensive trials to assess its efficacy and safety as a vaccine. Percutaneously administered *M. microti* vaccine was found to be safe but no more effective than *M. bovis* BCG (*12*). The low virulence and poor immunogenicity are due to several key genetic deletions, resulting in the inability to produce the strongly immunogenic T-cell antigens ESAT-6 and CFP-10 (*13*).

Several genotypes of *M. microti* have been recognized by spacer oligotyping (spoligotyping). The llama-type (presence of spacers 4–7, 23, 24, 26, 37, 38) and the vole-type (only 2 spacers, 37 and 38) have been well described; both types are involved in human infections (*5*,*7*). The international spoligotyping database (SpolDB4) (*14*) includes 40 *M. microti* strains, 37 of which are from the United Kingdom and Western Europe. Although there are no published reports of *M. microti* infections from the United States, 3 of the strains in SpolDB4 are from this country. *M. microti* strains yield broadly similar, high–copy number fingerprints by the insertion sequence *6110*–based restriction fragment length polymorphism method (IS*6110* RFLP) (*7*).

In the 12-year period from 1994 through 2005, we isolated *M. microti* from 4 humans and from 5 animals (2 cats, a llama, a badger, and a ferret). No clinical details were available for the animal cases. The animal and human cases were from different locations in Scotland. No epidemiologic links were apparent.

## The Patients

Patient 1 was a 41-year-old woman in whom sputum smear–positive tuberculosis was diagnosed in 2001. She was treated with isoniazid, rifampin, ethambutol, and pyrazinamide for 2 months and for 4 months more with rifampin and isoniazid. She made good clinical progress, but sputum samples remained positive for acid-fast bacilli (AFB), although cultures were negative. She was re-treated with isoniazid, rifampin, ethambutol, and pyrazinamide for 6 months. She became sputum negative and remained clinically well at her 6-month follow-up visit. She was not immunocompromised. No other patients with tuberculosis were identified in contacts, and no relevant animal contact had occurred.

Patient 2 was a 39-year-old man for whom HIV was diagnosed in 2003, who had bilateral pulmonary consolidation. The patient lived on a farm. He was initially treated with co-trimoxazole for suspected *Pneumocystis carinii* infection, and rifampin, isoniazid, and pyrazinamide were added when AFB were seen in the sputum sample. The patient’s condition deteriorated, and he died despite this drug treatment and intensive therapy unit support. No other patients with tuberculosis were identified in connection with this case.

Patient 3 was a 76-year-old woman who had received a diagnosis of pulmonary tuberculosis in 2005. She made an uneventful recovery following standard therapy with isoniazid, rifampin, and ethambutol for 2 months, followed by rifampin and isoniazid for a further 4 months. She was not immunocompromised, and she reported no major animal contact. No cases of tuberculosis were identified in connection with this patient.

Patient 4 was a 45-year-old woman who was seen in 2005 for hemoptysis; a diagnosis of cavitating pulmonary tuberculosis was made. She received treatment with isoniazid, rifampin, ethambutol, and pyrazinamide for 2 months and rifampin and isoniazid for 4 months more. She remained unwell, with further hemoptysis, and a residual cavity was shown on chest x-ray. Chemotherapy was reintroduced. She was not known to be immunocompromised. She had a pet cat and a dog, both in good health. No cases of tuberculosis were identified in contacts.

The laboratory characteristics of the isolates are shown in Table 2. Biochemical tests were not possible because of sparse growth. Isolates were identified as *M. tuberculosis* complex by using the Accuprobe culture confirmation assay (GenProbe, San Diego, CA, USA), and species identification as *M. microti* was confirmed by spoligotyping. Since we do not perform drug susceptibility testing using solid media, only the 3 strains that grew well in liquid subculture were tested. Genotyping data on our isolates are summarized in Table 2 and the Figure.

**Table 2:** Laboratory features of *Mycobacterium microti* isolates from Scotland*†

Source	Specimen	Direct AFB	Growth on primary isolation				Drug susceptibility	Genotype
			Solid culture			Liquid culture		
			IUT	PYR		MB/MGIT		
Human 1	Sputum	Positive	–	+		–	Failed to grow in liquid cultures	Llama type SIT641 (spacers 4–7, 23,24, 37, and 38)
Human 2	Sputum	Positive (many)	–	+		–	Failed to grow in liquid cultures	Llama type SIT641
Human 3	Sputum	Positive (many)	–	–		+	Susceptible to R, I, E; resistant to P	Llama type SIT641
Human 4	Sputum	Positive (few)	+	+		–	Failed to grow in liquid culture	Llama type (spacers 4–7 and 23,24 only)
Cat 1	Tissue/lymph node	Negative	–	+		–	Failed to grow in liquid cultures	Llama type SIT641
Badger	Tissue/lung	Strongly positive	+	+		±	Inadequate growth	Vole type SIT 539 (spacers 37 and 38)
Cat 2	Tissue/lymph node	Negative	–	+		–	Susceptible to R,I,E,P (grew on liquid subculture)	Vole type SIT539
Llama	Tissue/lung	Positive	+	+		–	Failed to grow in liquid culture	Llama type SIT641
Ferret	Tissue	Positive	±	±		±	Susceptible to R,I,E,P	Not tested

*AFB, acid-fast bacilli; IUT, International Union Against Tuberculosis formulation of solid egg medium; PYR, IUT medium with pyruvate supplementation; MB, MBBact, Biomerieux, Basingstoke, United Kingdom; MGIT, Mycobacteria Growth Indicator Tube, Becton Dickinson; R,I,E,P, rifampin, isoniazid, ethambutol, pyrazinamide; SIT, Spoligo-International-Type. †SIT numbers: designations in International spoligotyping database (Spol DB4) (*14*).

**Figure:**
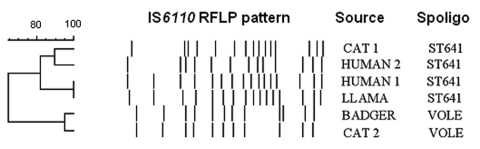
Comparison of the restriction fragment length polymorphism patterns of *Mycobacterium microti* strains from Scotland. Spoligo, spoligotyping.

## Conclusions

*M. microti* infection is widespread in wild small rodent populations in the United Kingdom (*2*). There are sporadic reports, all from the United Kingdom and Western Europe, of *M. microti* infection in other mammals. Certain animals, such as cats (*4*,*5*) and New World camelids domesticated in Europe (*6*), seem to be particularly susceptible. The reported animal cases have all been detected in clinical veterinary practice and are unlikely to reflect the true field incidence. Difficulties with laboratory diagnosis probably further contribute to the underestimation of the incidence. *M. microti* grows poorly on traditional solid egg media, and modern automated liquid culture techniques do not seem to yield better results. Moreover, even when a mycobacterial infection is diagnosed, routine veterinary diagnostic procedures often do not identify the mycobacterium to species level. It is likely also that known animal cases are not all formally reported in the literature.

The transmission of *M. microti* to pets, particularly cats, is of particular concern. Cats are assumed to acquire the *M. microti* infection from infected wild rodents, but this assumption is not supported by the genotyping evidence. Most of the strains isolated from cats are genotypically very distinct from wild rodent strains, as shown in our cases and in the literature (*5*). Very little is known about the incidence and ecology of *M. microti* infection in farm and domestic animals.

Many of the human patients with *M. microti* infection appear to have no immunologic deficits (3 of our 4 patients and 3 of the 8 published cases for which relevant clinical details were available). However, inherited defects of interleukin receptor function are known to specifically predispose to intracellular infections, particularly mycobacterial infection (*15*). Therefore, some persons with apparently normal immunity infected with *M. microti* may in fact have undetected specific immune defects.

Human-to-human transmission of *M. microti* infection seems rare. In the single instance in which this possibility is moot, the secondary cases all occurred in the same mice-infested household (*10*).

Extensive trials of *M. microti* as a vaccine suggest that it lacks virulence for humans with normal immunity. However, it remains a potential threat to the substantial pool of persons with compromised immunity, including the unknown number who may have genetic defects specifically predisposing to mycobacterial infections**.**
